# Live and fixed imaging of translation sites at single mRNA resolution in the *Drosophila* embryo

**DOI:** 10.1016/j.xpro.2021.100812

**Published:** 2021-09-13

**Authors:** Daisy J. Vinter, Caroline Hoppe, Hilary L. Ashe

**Affiliations:** 1 Faculty of Biology, Medicine and Health, University of Manchester, Manchester M13 9PT, UK

**Keywords:** Cell Biology, Developmental biology, Gene Expression, In Situ Hybridization, Microscopy, Model Organisms, Molecular Biology, Single-molecule Assays

## Abstract

Significant regulation of gene expression is mediated at the translation level. Here, we describe protocols for imaging and analysis of translation at single mRNA resolution in both fixed and living *Drosophila* embryos. These protocols use the SunTag system, in which the protein of interest is visualized by inserting a peptide array that is recognized by a single chain antibody. Simultaneous detection of individual mRNAs using the MS2/MCP system or by smFISH allows translation sites to be identified and quantified.

For complete information on the generation and use of this protocol, please refer to Vinter et al. (2021).

## Highlights

• Visualization of translation of single mRNAs in *Drosophila* using SunTag labeling
• Fixed embryo analysis reveals the mRNA translation efficiency in each expressing cell
• Live SunTag confocal imaging uncovers mRNA translation dynamics
• Image analysis pipelines allow quantitative and temporal information to be extracted

## Graphical abstract

**Figure:**
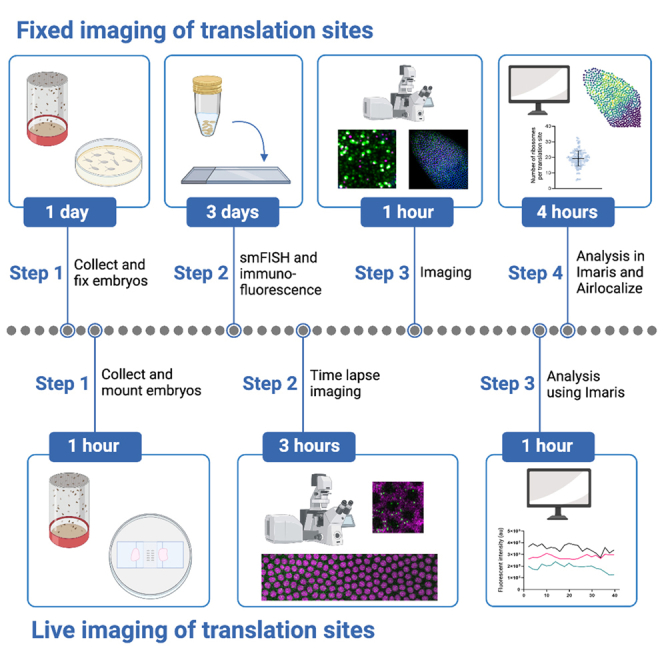

## Before you begin

### Experimental design considerations

**Figure 1:**
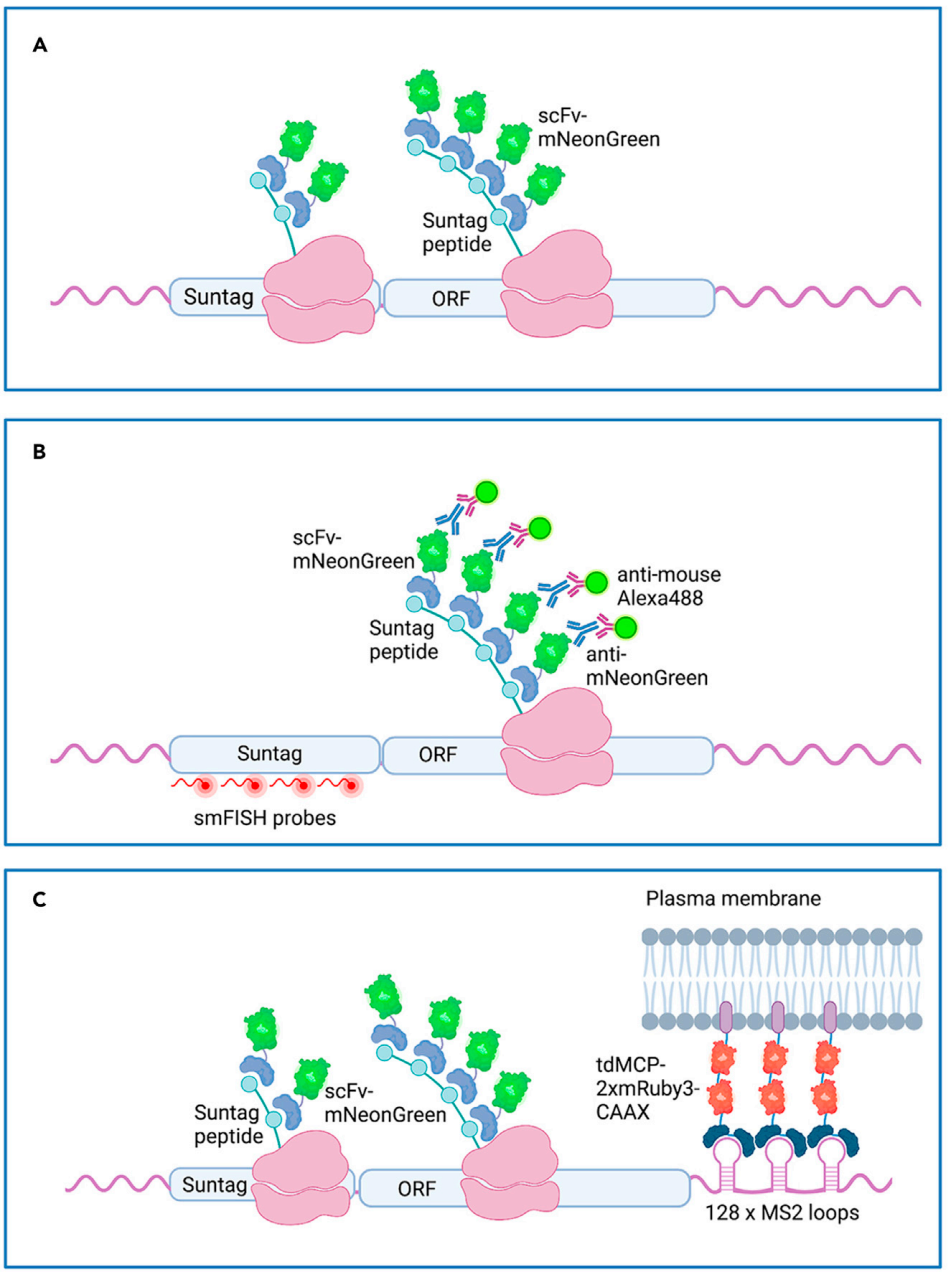
Overview of the SunTag imaging system (A) Schematic showing the SunTag system used to detect translation sites in live embryos. scFv-mNeonGreen binds the SunTag peptides as they are translated by the ribosome. (B) Schematic showing the SunTag detection used in fixed embryos. smFISH probes complementary to the SunTag sequences are used to identify individual mRNAs, and anti-mNeonGreen antibodies are used to detect scFv-mNeonGreen. (C) Schematic showing the SunTag system used for imaging of single translation sites in live embryos. 128 MS2 loops are inserted into the 3′UTR. tdMCP-2xmRuby3-CAAX binds the MS2 loops and the CAAX sequence anchors the translating mRNA complex to the plasma membrane.

Introduction of the SunTag array into the protein of interest (POI), and subsequent binding of a single chain antibody-fluorescent protein fusion (scFv-FP) to the nascent SunTag peptides (Figure 1A), allows translation to be visualized in the *Drosophila* embryo (Dufourt et al., 2021; Vinter et al., 2021). Insertion of SunTag at the start of the coding sequence enables rapid visualization of translation sites and maximizes their signal, facilitating early detection and quantitation.

Single molecule fluorescent in situ hybridization (smFISH) is used with SunTag labeling (Figure 1B) to visualize translation of individual mRNAs in fixed embryos, whereas the MS2/MCP system for mRNA detection is exploited for live imaging of translation. Insertion of 128 MS2 copies gives the required sensitivity for single mRNA detection (Vinter et al. 2021; Dufourt et al. 2021). Introduction of the MS2 sequences into the 3′UTR is optimal, as stem loops in the 5′UTR can disrupt ribosome scanning and reduce translation efficiency (Palam et al., 2011; Vattem and Wek, 2004). If inserted into the coding sequence, the MS2 loops will be unwound by the translating ribosome, which will displace bound MCP and disrupt labeling of the mRNA (Halstead et al., 2015). As 3′UTR sequences are known to bind regulatory factors (Mayr, 2017), insertion of the MS2 loops at the beginning of the 3′UTR will leave it otherwise intact, reducing the potential of the MS2 loops to disrupt regulation. To track and image mRNAs for extended periods of time (minutes), an MCP-FP-CAAX fusion is used, which reduces mRNA movement via anchoring to the plasma membrane (Vinter et al., 2021; Yan et al., 2016) (Figure 1C).

We describe methods for imaging mRNA translation in fixed samples (Section 1: Fixed imaging of individual mRNAs and translation sites) and live imaging of both translation sites and anchored single mRNAs (Section 2: Live imaging of translation sites). Analysis pipelines for detecting mRNAs and determining their translation efficiency and ribosome density are also presented. The protocols described here have been optimized for the *scFv-mNeonGreen* (*scFv-mNG*), *SunTag-hunchback(hb)(+/-128xMS2)* and *tdMCP-mRuby3-CAAX* fly lines (Vinter et al. 2021), which we use as examples throughout. However, the protocols and analysis pipelines can be extended to any SunTag-POI or scFv-FP.

### Generation of the SunTag-POI fly line(s)

**Timing: ∼8 or ∼12 weeks to generate a homozygous transgenic fly stock or CRISPR line, respectively**

1. Generate a fly line expressing the protein of interest with the SunTag sequences (SunTag-POI) either by genome editing or by introducing a transgene. MS2 sequences should also be inserted in the 3′UTR if live imaging of the mRNAs is required.

***Note:*** When using a SunTag-POI transgene it is important to include the coding sequence and both UTRs to recapitulate regulation of the endogenous mRNA more accurately.

***Note:*** Generation of SunTag-POI transgenics by phiC31-mediated integration (Bischof et al., 2007; Fish et al., 2007) allows an appropriate landing site to be used to avoid position effects. This is especially important when testing, for example, mutant transgenes in follow up experiments.

***Note:*** Detailed CRISPR protocols have been described (Hoppe and Ashe, 2021a; Yu et al., 2021), including methods for scarless editing (Lamb et al., 2017; Nyberg et al., 2020).

## Key resources table

**Table:** 

REAGENT or RESOURCE	SOURCE	IDENTIFIER
**Antibodies**		

Mouse anti-mNeonGreen	Chromotek	RRID: AB_2827566
Donkey anti-Mouse IgG (H+L) Highly Cross-Adsorbed Secondary Antibody, AlexaFluor 488	Thermo Fisher Scientific	RRID: AB_141607

**Chemicals, peptides, and recombinant proteins**		

Methanol, Certified AR for Analysis	Fisher Scientific	M/4000/17
Bleach	Banner	Cat #: 1190012
Halocarbon Oil 700	Sigma	Cat #: H8898
Halocarbon Oil 27	Sigma	Cat #: H8773
DAPI	Thermo Fisher Scientific	Cat #: D1306
SSC Buffer 20**×** Concentrate	Sigma	Cat #: S6639
Formamide, Spectrophotometric Grade, ≥99%	Honeywell	Cat #: 295876
Formaldehyde Solution	Thermo Fisher Scientific	Cat #: 15627860
Heptane	Sigma	Cat #: H2198
Tween-20	Sigma	Cat #: P9416
Phosphate buffered saline (PBS) tablets	Sigma	Cat #: P4417
Dextran sulfate sodium salt from *Leuconostoc* spp.	Sigma	Cat #: D8906
ProLong Diamond Antifade Mountant	Thermo Fisher	Cat #: P36961
TE, pH 8.0, RNase-free	Thermo Fisher	Cat #: AM9849
Triton X-100	Sigma	Cat #: X100
Ethylene glycol-bis(2-aminoethylether)-*N,N,N′,N′*-tetraacetic acid (EGTA)	Sigma	Cat #: E3889
Western Blocking Reagent, Solution	Sigma	Cat #: 11921673001

**Experimental models: organisms/strains**		

*D.melanogasterw*^*∗*^ ; *nos-scFv-mNeongreen*	(Vinter et al., 2021)	Available on request
*D.melanogasterw*^*∗*^ ;*; hbP2>SunTag-hb*	(Vinter et al., 2021)	Available on request
*D.melanogasterw*^*∗*^ ;; *hbP2>SunTag-hb-128xMS2*	(Vinter et al., 2021)	Available on request
*D.melanogasterw*^*∗*^ ;*;nos-tdMCP-mRuby3-CAAX*	(Vinter et al., 2021)	Available on request
*D.melanogasterHis-2Av-RFP*	Bloomington Drosophila Stock Center	BL23650

**Oligonucleotides**		

SunTag Stellaris® smFISH probes (Quasar 570 tagged at the 3′ end)	Biosearch Technologies	See Table 1

**Deposited data**		

Example datasets	This study	Fixed data: Mendeley data https://doi.org/10.17632/3h73rbvbt9.1 Live data: Mendeley data https://doi.org/10.17632/5vs879zvz9.1

**Software and algorithms**		

Imaris 9.2	Bitplane	RRID:SCR_007370
Huygens Professional Deconvolution software	SVI	RRID:SCR_014237-
Software for assignment to nucleus ‘sass’	(Hoppe et al., 2020; Vinter et al., 2021)	https://github.com/TMinchington/sass, RRID:SCR_018797
Software for removal of nuclear spots	This study; (Vinter et al., 2021)	https://github.com/DaisyVinter/StarProtocols_Vinter_2021
Software for calculation of ribosome number	This study; (Vinter et al., 2021)	https://github.com/DaisyVinter/StarProtocols_Vinter_2021
Airlocalize	Trcek et al. 2017	Available on request from T. Lionnet
Python 3.7	N/A	N/A
Fiji	(Schindelin, 2012)	RRID: SCR 002285

**Other**		

Coverslips Nr. 1, 18 **×** 18 mm	Deltalab	Cat #: D101818
Coverslips Nr. 0, 18 **×** 18 mm	Scientific Laboratory Supplies	Cat #: MIC3100
Coverslips Nr. 1, 24 **×** 50 mm	Deltalab	Cat #: D102450
Slides, 26 **×** 76 mm	Deltalab	Cat #: D100004
Scintillation vial, glass, cap, 20 mL	Thermo Fisher Scientific	Cat #: 03-337-15
V-Vials with solid-top screw cap, capacity 0.3 mL	Sigma	Cat #: Z115053-12EA
Lumox dish 50, cell culture dish	Sarstedt AG & Co	Cat #: 94.6077.305
Yellow double-sided plastic tape, 25 mm **×** 20 m	RS Components	Cat #: 555-033
Packaging tape	Tesa	Cat #: 4124

## Materials and equipment

**Table:** 

Embryo washing solution	Final concentration	Amount
NaCl	4 g/L	4 g
10% Triton X-100	0.03%	3 mL
ddH_2_O	n/a	997 mL
**Total**	**n/a**	**1 L**

Embryo washing solution can be kept for long periods at 20^o^C.

**Table:** 

Fixing buffer	Final concentration	Amount
10 **×** PBS	1.33 **×**	13.3 mL
0.333 M EGTA (pH 8.0)	0.067M	20 mL
ddH_2_O	n/a	66.7 mL
**Total**	**n/a**	**100 mL**

Fixing buffer can be stored at 20^o^C for extended periods.

**Table:** 

Stellaris hybridization buffer	Final concentration	Amount
Dextran Sulfate	100mg/mL	300 mg
Formamide	10%	300 μL
20**×** SSC	2**×**	300 μL
Nuclease free H_2_O	n/a	2.4 mL
**Total**	**n/a**	**3 mL**

This will make enough hybridization buffer for one sample. Excess hybridization buffer can be stored at −20°C but should only be freeze-thawed a maximum of twice.

**Table:** 

Stellaris Wash buffer	Final concentration	Amount
Formamide	n/a	500 μL
20**×** SSC	2**×**	500 μL
Nuclease free H_2_O	n/a	4 mL
**Total**	**n/a**	**5 mL**

This will make enough wash buffer for one sample. Make up fresh for each experiment and store in the fridge between day 1 and 2 of smFISH protocol.

## Step-by-step method details

### Section 1: Fixed imaging of individual mRNAs and translation sites

**Figure 2:**
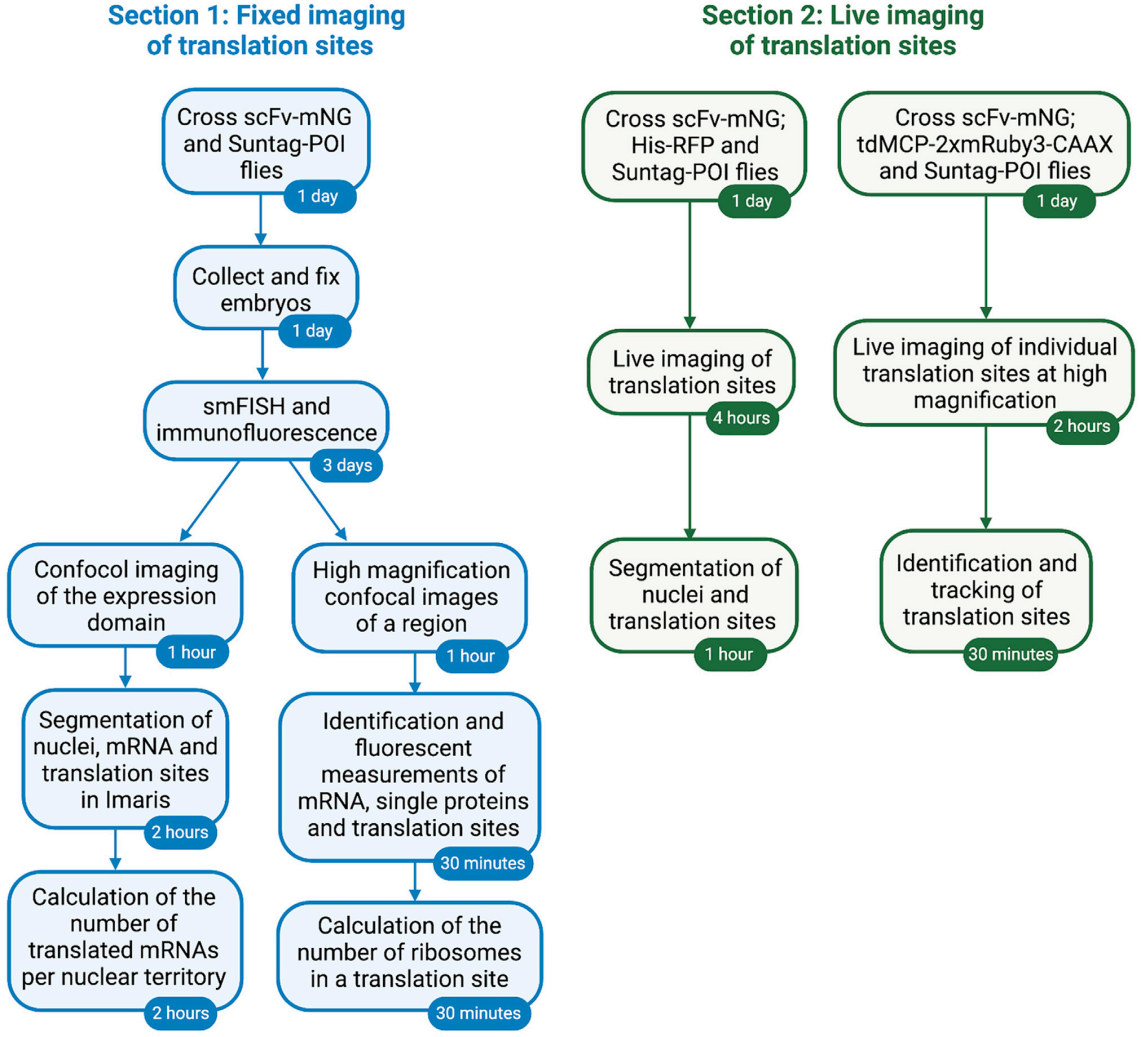
Overview of the protocols for visualizing translation sites The steps associated with the detection and analysis of translation sites in fixed and live embryos are shown, with the relevant timings.

This section describes how to collect and fix embryos for detection of *SunTag-hb* mRNAs and their translation sites using a combination of smFISH and immunofluorescence (Figure 2). Methods to calculate total mRNA number, the number of mRNAs that are translated, and ribosome number in the translation sites are also described.

#### Set up embryo collection cages

**Timing: 1–3 days**

In this step, flies are crossed to allow the collection of embryos with maternally expressed *scFv-mNG* and zygotically expressed *SunTag-hb*.

1. Collect virgin females homozygous for the *nos-scFv-mNG* transgene and males homozygous for the *SunTag-hb* transgene.

***Note:*** This cross will produce embryos that zygotically express one copy of *SunTag-hb*. If visualization of translation of the full complement of mRNAs from both alleles is required, balanced stocks can be generated to allow the collection of embryos that maternally express *scFv-mNG* and are homozygous for the *SunTag-POI* transgene/CRISPR modified locus.

***Note:*** Control embryos can also be collected from stocks carrying only the *SunTag-POI* or the *scFv-FP* transgene if required.

***Note:*** Fly lines maternally expressing *scFv-sfGFP* and *scFv-mScarlet* (Dufourt et al., 2021) are also available. There are also *UASp-scFv-sfGFP* (Lu et al., 2016) and *UASp-scFv-GFP-NLS* (Formicola et al., 2021) stocks that are compatible with the GAL4/UAS system, allowing translation to be imaged in a cell type specific manner at later developmental stages (Formicola et al., 2021).

2. Transfer at least 100 females and 50 males to a collection cage with an apple juice agar plate and yeast paste.

***Note:*** Increasing the number of flies in the collection cage will increase the number of embryos collected.

#### Collect and fix embryos

**Timing: 1 day**

In this step, embryos are collected and fixed for use in smFISH and immunofluorescence to allow *SunTag-hb* translation sites to be visualized.

3. Change the plate on the collection cage to a fresh apple juice agar plate with yeast and collect embryos at 25°C for the desired length of time. For collection of a range of nuclear cycle (nc) 12 – nc14 embryos, let the flies lay embryos for 3 h, then remove the plate and age the embryos for 1 h at 25°C.

***Note:*** We do not recommend shifting collection temperatures to change the speed of development, because translation is temperature sensitive.

4. Dechorionate the embryos by applying 50% domestic bleach to the plate and incubating for 2 min.
5. Transfer the embryos to a sieve by washing them off the plate with water or embryo washing solution.
  a. When in the sieve, wash thoroughly with water or embryo washing solution until no bleach remains.
6. Transfer embryos into a glass scintillation vial containing 3 mL fixing buffer.
7. Add 4 mL heptane and 1 mL formaldehyde and shake at 300 rpm for 20 min.
8. Remove the liquid layer at the bottom of the vial with a plastic Pasteur pipette.

***Note:*** Take care not to lose any embryos while removing the liquid. It is better to retain embryos than to remove all of the liquid layer.

9. Devitellinize the embryos.
  a. Add 8 mL of methanol to the glass vial.
  b. Shake the vial vigorously for 30 s and vortex for 30 s.
  c. Remove the liquid and repeat at least twice more until the liquid is clear and has no floating membrane.

**CRITICAL:** Ensure that the devitellinization step is complete so that the staining and imaging are optimal. Use more methanol washes and shaking if necessary.

10. Transfer the embryos in methanol to a 1.5 mL eppendorf tube. Remove as much methanol as possible and replace with fresh methanol. Embryos can then be used for smFISH and immunofluorescence or kept in methanol at −20°C.

**Pause point:** Embryos can be kept at −20°C for at least three months without a reduction in the quality of the smFISH and immunofluorescence staining.

***Note:*** Depending on the number of flies in the collection cage and the efficiency of laying, embryo collections may need to be carried out on multiple days to collect enough embryos for smFISH and immunofluorescence.

#### smFISH and immunofluorescence

**Timing: 3 days**

In this step, embryos are stained with *SunTag* smFISH probes to recognize individual mRNAs and an anti-mNeonGreen antibody to identify the scFv-mNG signal (Figure 1B). Steps are performed at 20^o^C unless stated otherwise.

Day 1:

11. Prepare the SunTag smFISH probes (Table 1).
  a. Reconstitute the lyophilized probes in 400μL RNase-free TE and mix by pipetting. This will produce a probe stock with a concentration of 12.5μM.
  b. Make 20μL aliquots of the dissolved probes. Store at −20°C and protect from the light.

**Table 1:** Sequences of the SunTag stellaris probes

Probe name	Probe sequence (5′-3′)	Probe name	Probe sequence (5′-3′)
SunTag_1	Ccacttcgttctcaagatga	SunTag_25	cgcgacttcgttctctaaat
SunTag_2	gccagaacctttcttaagac	SunTag_26	ttcgataagagttcttcgcc
SunTag_3	ttgaaagcagttcttctcca	SunTag_27	ctcattttcgaggtggtagt
SunTag_4	ctcattttccaggtggtaat	SunTag_28	gtggtagttcttgctcaag
SunTag_5	aatttttgctcagcaactcc	SunTag_29	attcttgctgagcaattcct
SunTag_6	ttctttagtcgtgctacttc	SunTag_30	cgacttcgttctccaaatga
SunTag_7	tttcgagagtaactcctcac	SunTag_31	tttactcaacaattcctccc
SunTag_8	ccacttcgttttcgagatga	SunTag_32	cgacttcattttccaagtgg
SunTag_9	gataatagctcttctccaga	SunTag_33	ttgctcaataactcttcgcc
SunTag_10	tcattttcgaggtggtagtt	SunTag_34	ttcgttctccaagtggtaat
SunTag_11	acttcccttttttaagcgtg	SunTag_35	agttcttcgataagagctcc
SunTag_12	tcttggatagtagctcttca	SunTag_36	gcgacttcattctctaagtg
SunTag_13	ctacctcgttctcaagatga	SunTag_37	agtggtagttcttgctcaag
SunTag_14	tagttcttcgagagcagttc	SunTag_38	ttagatagtaactcttcccc
SunTag_15	gatcccttttttaatcgagc	SunTag_39	cctcgttctcgagatgataa
SunTag_16	tgaaagtagttcctcaccac	SunTag_40	gatagttcttcgacaggagt
SunTag_17	cttcgttttcgaggtggtaa	SunTag_41	cctttttaagtcttgcaacc
SunTag_18	ccctgaacctttctttaatc	SunTag_42	ttcttactgagtagttcctc
SunTag_19	tactcagtaattcttcaccc	SunTag_43	tcctgatcctttcttcaaac
SunTag_20	ctacctcattttccagatga	SunTag_44	cttttgagagcagttcttcg
SunTag_21	tttcgatagcaactcttcgc	SunTag_45	gcaacctcattttccaaatg
SunTag_22	tttttgagcctagcaacttc	SunTag_46	tgccacttcccttttttaaa
SunTag_23	agtggtagtttttcgagagc	SunTag_47	tttcgacagaagttcctcac
SunTag_24	tttgctcaataactcctcgc	SunTag_48	taagtcgggctacttcattc
12. Transfer the embryos to a glass V-Vial using a pipette with a cut tip. A volume of 50 μL of embryos per stain is optimal because some embryos are lost throughout the staining process.
13. Perform washes with 500 μL of the following solutions.
  a. Rock in 50:50 methanol:PBT (1 **×** PBS + 0.1% Tween-20) for 5 min.
  b. Rock in PBT for 10 min. Repeat 3 more times.
  c. Rock in 50% PBT, 50% Stellaris wash buffer for 10 min.
  d. Rock twice in Stellaris wash buffer for 5 min.
  e. Add 500 μL Stellaris wash buffer and incubate for 2 h in a 37°C water bath.
14. Stain with SunTag smFISH probes.
  a. Dilute the SunTag probes to a final concentration of 50 nM in 300 μL Stellaris hybridization buffer.
  b. Incubate the embryos in the probe mixture in a 37°C water bath for 12–18 h.

**CRITICAL:** Incubate the embryos in the probe mixture for at least 12 h. Keep the Stellaris wash and hybridization buffers at 4°C for the duration of the experiment; fresh Stellaris wash buffer should be made up for each experiment.

**CRITICAL:** After probe hybridization, embryos should be kept in the dark as far as possible. Cover the V-Vials with foil to maintain darkness.

***Note:*** Using SunTag probes has the advantage that they are generic for different SunTag-POI mRNAs. Moreover, detection is limited to mRNAs carrying SunTag sequences, excluding mRNAs transcribed from the endogenous/unmodified locus, which simplifies the analysis.

***Alternatives:*** SunTag smiFISH (single molecule inexpensive FISH) probes, which have specific sequences against the SunTag and a common flap sequence for the hybridization of fluorescently labelled probes (Tsanov et al., 2016), may be used to reduce costs. In this case, incubate smiFISH probes with fluorescently tagged flap probes prior to dilution in Stellaris hybridization buffer (Calvo et al., 2021). We have not tested smiFISH probes against SunTag sequences, but have used them to detect other individual mRNAs (Hoppe et al, unpublished).

Day 2:

**CRITICAL:** Maintain the embryos in darkness as much as possible on Day 2.

15. Pre-warm Stellaris hybridization and wash buffers to 37°C in a water bath.
16. Perform washes to remove unbound probes with 500 μL of the following solutions.
  a. Wash embryos in Stellaris hybridization buffer for 30 min in a 37°C water bath.
  b. Rinse with Stellaris wash buffer.
  c. Wash embryos in Stellaris wash buffer for 15 min in a 37°C water bath. Repeat twice.
  d. Rock in Stellaris wash buffer for 15 min.
  e. Rock 3 times in PBT for 10 min.
  f. Rock in PBT + 1 **×** Western Blocking Reagent (WBR) for 30 min.
17. Incubate with the primary antibody.
  a. Dilute anti-mNeonGreen antibody (Chromotek, RRID: AB_2827566) 1:250 in PBT + 1 **×** WBR.
  b. Rock in the primary antibody solution at 4°C for 12–18 h.

***Alternatives:*** The primary antibody incubation can be shortened to 2 h at 20^o^C, allowing the protocol steps described for days 2 and 3 to be completed in a single day.

***Note:*** Not all antibodies are compatible with the smFISH protocol so new antibodies will have to be tested in combination with the smFISH protocol.

Day 3:

**CRITICAL:** Keep the embryos in the dark where possible on Day 3.

18. Perform washes to remove any unbound antibody with 500 μL of the following solutions.
  a. Rinse the embryos with PBT twice.
  b. Rock the embryos 4 times in PBT for 15 min.
  c. Rock the embryos in PBT + 1 **×** WBR for 30 min.
19. Incubate with the secondary antibody.
  a. Dilute anti-mouse AlexaFluor 488 (Thermo Fisher Scientific, RRID: AB_141607) at 1:250 in PBT + 1 **×** WBR.
  b. Rock the embryos in the secondary antibody solution for 2 h at 20^o^C.
20. Perform washes in 500 μL volumes.
  a. Rinse the embryos twice with PBT.
  b. Rock twice in PBT for 15 min.
  c. Dilute 500 μg/mL DAPI at 1:1000 in PBT. Rock embryos in DAPI solution for 15 min.
  d. Rock in PBT for 15 min.

***Alternatives:*** If using ProLong Diamond mountant with DAPI, omit the DAPI incubation step.

21. Mount embryos.
  a. Remove as much PBT as possible.
  b. Add approximately 3 drops of ProLong Diamond to the embryos.
  c. Use a glass pipette to draw up the embryos and pipette them onto a glass slide.
  d. Carefully cover with a Nr 1, 24 mm **×** 50 mm coverslip by lowering onto the slide with tweezers.
22. Dry the slides for 12–18 h at 20^o^C in the dark. The slides can then be used for imaging or can be stored at −20°C.

**Pause point:** Slides can be stored at −20°C for at least 3 months without loss of staining, but we recommend imaging the embryos as soon as possible.

#### Imaging

**Timing: ∼1 h per image, dependent on image size**

In this step, embryos are imaged using confocal microscopy. Images can be taken at the level of the expression domain, and the total numbers of mRNAs and translated mRNAs can be calculated. In addition, higher magnification images can be taken and used to calculate the number of ribosomes on each mRNA.

23. Image the stained embryos using confocal microscopy. 3 channels will be required, for SunTag smFISH, mNeonGreen staining, and DAPI (Troubleshooting 1). For analysis of translation along the anterior-posterior axis, align the axis diagonally to maximize the area of embryo imaged (see the example in Figure 6A) and for compatibility with axis calculation in the spots assignment script used in Step 34.
  a. For the example whole expression domain dataset, we used a Leica TCS SP8 AOBS inverted confocal microscope with 63**×**/1.40 HCX PL APO CS oil objective and 0.75**×** optical zoom, and white light laser set to 70%.
    i. Set the pinhole to 1 airy unit and scan speed to 400 Hz, with 2**×** line averaging and bit depth of 8. Acquire an image at 2048 x 2048 pixels, resulting in a pixel size of 0.12 μm x 0.12 μm, and z-step size of 300 nm, for 107 z-steps, giving an imaging depth of 31.8 μm. The z-depth should include the most apical to the most basal fluorescent signal in the SunTag smFISH and the scFv-mNG channels.
    ii. Collect the DAPI channel with 405 nm excitation (2%), using a PMT detector with collection set from 415 – 470 nm.
    iii. Collect the scFv-mNG (AlexaFluor 488) channel with 488 nm excitation (10%), using a hybrid detector with collection set from 500 – 540 nm.
    iv. Collect the SunTag smFISH (Quasar 570) channel with 548 nm excitation (15%) using a hybrid detector with collection set from 558 – 640 nm.

**CRITICAL:** Make sure the sample slide reaches 20^o^C before imaging.

***Alternatives:*** The expression domain can be aligned along the x or y axis, allowing cellular outputto be analyzed using the x or y position of nuclei.

***Note:*** For whole embryo/expression domain imaging, use an objective with magnification sufficient to capture the whole expression domain. We use a laser power for the SunTag smFISH channel which overexposes the transcription sites allowing individual mRNAs to be reliably imaged and detected by the analysis software. If the number of mRNAs within the transcription site is to be analyzed, a lower laser power must be used to prevent overexposure.

***Note:*** Imaging at least three biological replicates (embryos) is recommended as there will be cell-to-cell and embryo-to-embryo variation, especially when analyzing mutant genotypes. Images taken with the example settings will provide ∼250 (nc12) – 1000 (nc14) nuclei per embryo.

24. Image a high magnification region of a stained embryo for calculation of ribosome number.
  a. For the example ribosome number imaging dataset, we used a Leica TCS SP8 AOBS inverted confocal microscope with 100**×**/1.40 HCX PL APO oil objective and 3**×** optical zoom, and white light laser set to 70%.
    i. Set the pinhole to 0.65 airy unit and scan speed to 400 Hz, with 4**×** line averaging and bit depth of 8. Acquire an image at 4096 x 4096 pixels, resulting in a pixel size of 0.009 μm x 0.009 μm, and z-step size of 200 nm. The example dataset contains 64 slices, resulting in a total imaging depth of 12.6 μm in the z direction.
    ii. Collect the DAPI channel with 405 nm excitation (11%), using a PMT detector with collection set from 415 – 470 nm.
    iii. Collect the scFv-mNG (AlexaFluor 488) channel with 488 nm excitation (12%), using a hybrid detector with collection set from 500 – 540 nm, and gating of 1–6 μs.
    iv. Collect the SunTag smFISH (Quasar 570) channel with 548 nm excitation (20%) using a hybrid detector with collection set from 558 – 640 nm, and gating of 1–6 μs.

**CRITICAL:** To calculate the number of ribosomes, translation sites must not be overexposed in order to capture the total amount of fluorescence. Ensure there are no overexposed pixels in the scFv-mNG channel.

25. **(Optional)** Deconvolve images using Huygens Professional Deconvolution Software (SVI).

***Note:*** We deconvolve the images to improve the signal-to-noise ratio. However, this is not essential if deconvolution is not preferred, and the raw fluorescence will be sufficient to analyze the datasets.

#### Analysis of the number of translated mRNAs

**Timing: ∼5 h per image, dependent on image size and the number of mRNAs, including the time for running the assignment script.**

**Figure 3:**
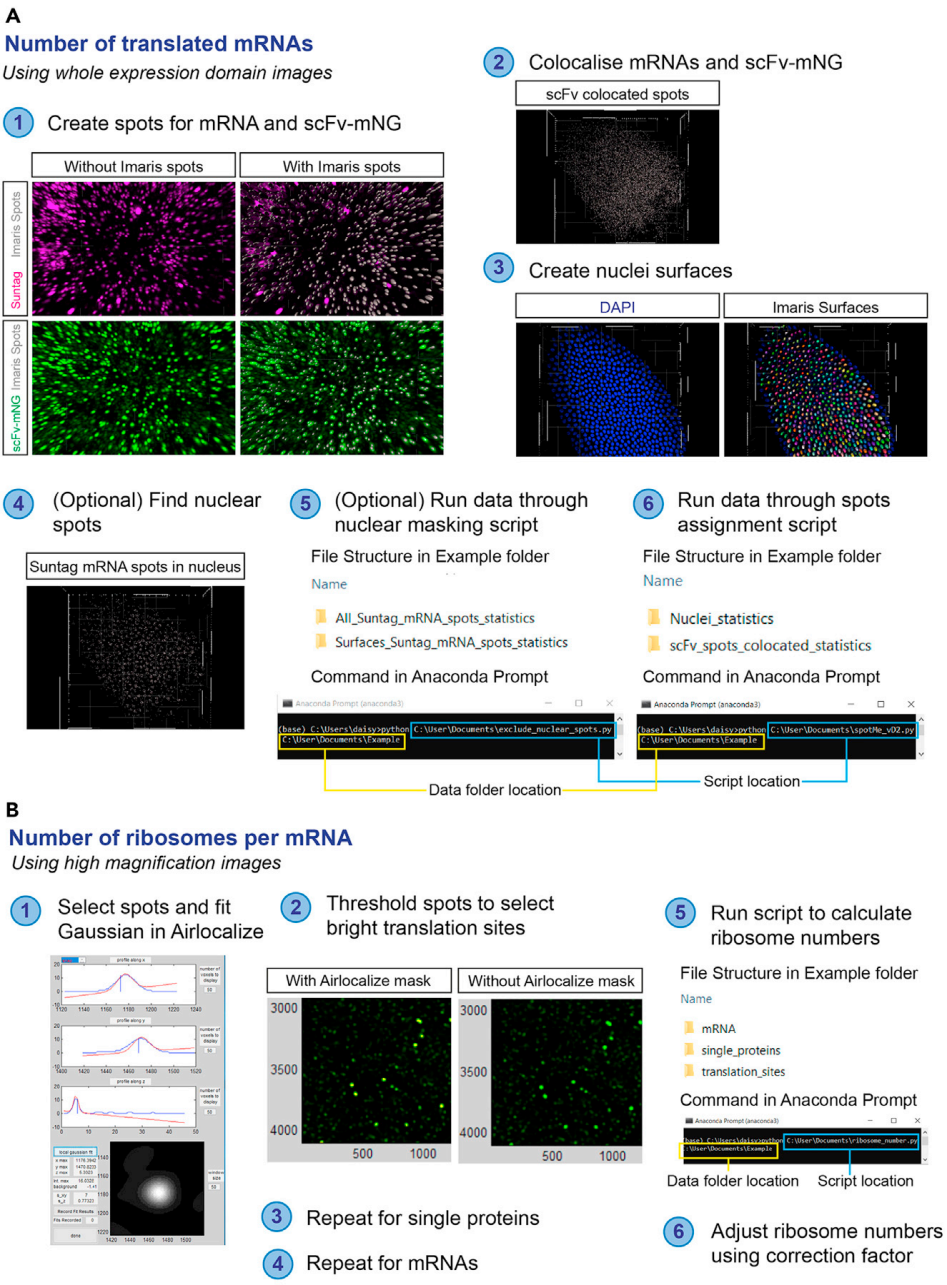
Overview of the analysis of translation sites from fixed embryo images (A) The steps show how the mRNAs and scFv-mNG signals are detected, colocalized to identify translation sites, then assigned to the nearest nucleus. (B) The steps show how to analyze the high magnification images to calculate the number of ribosomes per mRNA.

In these steps, the whole expression domain images are analyzed using Imaris 9.2 and a custom python script is used to quantify the total mRNA number and the number that are translated (Figure 3A).

26. Load the image into Imaris 9.2. Most common image formats can be opened in Imaris 9.2. If using a later version of Imaris, run the image through the Imaris File Converter to convert the image to .ims format.
27. Using the Imaris ‘spots’ function, create spots to identify the mRNAs based on the Suntag smFISH signals (Figure 3A, Step 1). Follow the steps in the ‘spots’ creation wizard.
  a. Leave the first screen as default settings.
  b. Select the channel used for SunTag smFISH.
  c. Estimate the XY diameter by moving to ‘Slice’ view and measuring the diameter of individual mRNA foci. We find a diameter of 0.6 μm to be optimal but this will vary depending on the imaging settings.
  d. Select ‘Model PSF-elongation along Z-axis’ and ‘Background Subtraction’.
  e. To remove transcription sites from analysis, set the ‘Filter Type’ to ‘Intensity Sum Ch=X Img=1’ where Channel X is the channel used for SunTag smFISH that is currently being worked on. Select only spots which are low in fluorescence (the majority of the spots), removing the tail of spots with high fluorescence. This will remove any nascent transcription sites (brighter than individual mRNAs) from the analysis.
  f. Add a filter with ‘Filter Type’ set to ‘Quality’. Drag the filter until all mRNA spots are selected.
  g. Complete the spots wizard.
  h. Name the spots ‘SunTag mRNA spots’.

**CRITICAL:** It can be difficult to identify spots by eye when there is a large number of mRNAs in one area. We find it is simplest to change the quality filter while looking at a region with less mRNAs, for example the edge of an expression domain. Ensure that each mRNA molecule in this region is selected without any false positives (Figure 3A, Step 1). Check that no excess spots are selected by toggling on and off the ‘Spots’ view under ‘Scene’.

28. Use the Imaris ‘spots’ function to identify the potential translation sites based on the scFv-mNG signal (Figure 3A, Step 1). Follow the steps in the ‘spots’ creation wizard.
  a. Leave the first screen as the default settings.
  b. Select the channel used for scFv-mNG.
  c. Estimate the XY diameter by moving to ‘Slice’ view and measuring the diameter of individual scFv-mNG foci. We find a diameter of 0.5 μm to be optimal but this will vary depending on the imaging settings and the size of the scFv-mNG translation sites, which depends on translation efficiency. Note that the scFv-mNG foci will vary in size – use the diameter of the smaller spots to ensure all are captured.
  d. Select ‘Model PSF-elongation along Z-axis’ and ‘Background Subtraction’.
  e. Set the ‘Filter Type’ to ‘Quality’. Move the filter until all scFv-mNG spots are captured.
  f. Complete the spots wizard.
  g. Name the spots ‘scFv spots’.

**CRITICAL:** The amount of background in the scFv-mNG channel will increase as translation occurs, due to increased numbers of SunTag-test proteins that have been translated and released, still bound by scFv-mNG. This can make distinguishing scFv-mNG translation sites from background more difficult. In this case, we recommend capturing all the translation sites (identifiable visually by colocalization with mRNAs) as spots. While most background signals will be avoided, this threshold may cause Imaris to detect some background spots (for example, in the nucleus) (Figure 3A, Step 1). However, as we call scFv-mNG signals as translation sites only if they have an overlapping smFISH signal, the colocalization step will eliminate scFv-mNG background spots that lack a colocalized mRNA (Troubleshooting 2).

29. Colocalize the SunTag mRNA and scFv spots to identify translation sites based on the presence of both an smFISH and scFv-mNG signal (Figure 3A, Step 2).
  a. In the ‘X-tensions’ tab, select the ‘Co-localize spots’ X-tension.
  b. Select the two spots masks to co-localize –‘SunTag mRNA spots’ and ‘scFv spots’.
  c. Input the threshold to call co-localization. We use a threshold of 0.3 (300 nm), which was chosen based on our data but is also consistent with the distance used in a previous SunTag study (Wu et al., 2016).
30. Using the Imaris ‘Surfaces’ function, create surfaces to identify nuclei. Nuclei will be used to remove nuclear mRNAs from the analysis (optional) and to assign mRNAs to their closest nucleus. Follow the steps in the ‘surfaces’ creation wizard (Figure 3A, Step 3).
  a. Leave the first screen as the default settings.
  b. Select the channel used for the nuclear stain.
  c. Select ‘Smooth’ and set ‘Surfaces Detail’ to 0.5 μm.
  d. Estimate the nuclear size by moving to ‘Slice’ view and measuring the diameter of individual nuclei.
  e. Select ‘Background Subtraction (Local Contrast)’ and set ‘Diameter of largest Sphere which fits into the Object’ as the value measured in step d.
  f. Change the Threshold (Background Subtraction) filter until all nuclei are covered.
  g. Select ‘Enable’ to Split touching Objects. Set the ‘Seed Points Diameter’ to the value used for ‘Diameter of largest Sphere which fits into the Object’.
  h. Set the ‘Filter Type’ to ‘Quality’ and drag until all nuclei have an object.
  i. Complete the surfaces wizard.
  j. Name the surfaces ‘nuclei’.
31. **(Optional)** Identify mRNAs inside the nucleus and check if any translation sites have been assigned inside the nucleus (Figure 3A, Step 4). This step is used to remove nuclear mRNAs so that the proportion of translated mRNAs is only calculated based on the total number of cytoplasmic mRNAs, which are accessible to the translation machinery. As there should be no translation sites identified in the nucleus, this step also functions as a control to check whether there are any false positive translation sites.
  a. Select ‘Suntag mRNA spots’ and run the Imaris X-tension ‘Split spots into surface objects’.
  b. Select ‘scFv spots co-located’ and run the Imaris X-tension ‘Split spots into surface objects’.
32. Export the Imaris data.
  a. Export the ‘SunTag mRNA spots’ data from step 27. This represents the total mRNA data. If removal of nuclear mRNAs is required, also export the ‘Suntag mRNA spots inside Surfaces’ folder from step 31a.
  b. Export the ‘scFv spots co-located’ data, the translated mRNA data. If removal of any false positive translation sites is needed, also export the ‘scFv spots co-located inside Surfaces’ folder.
  c. Export the ‘nuclei’ data.

**CRITICAL:** Ensure that ‘Position’ and all files starting with ‘Intensity’ are included in the data exported for ‘Suntag mRNA spots’ and ‘scFv spots co-located’, and that ‘Position’ and all files starting with ‘Ellipsoid Axis’ are included in the data exported for ‘nuclei’.

33. **(Optional)** If removal of nuclear mRNAs is required for the analysis, run the data through the remove_nuclear.py script (https://github.com/DaisyVinter/StarProtocols_Vinter_2021) (Figure 3A, Step 5).
  a. Create a folder containing the ‘Suntag mRNA spots’ and ‘Suntag mRNA spots inside Surfaces’ data.
  b. In Anaconda Prompt, type: python. Drag and drop the remove_nuclear.py script, and then drag and drop the folder created in step a.
  c. The script will create two folders in the folder created in step a – ‘Suntag mRNA cytoplasmic spots’ and ‘Suntag mRNA nuclear spots’, containing the separate Imaris outputs for cytoplasmic and nuclear mRNAs. Use the ‘Suntag mRNA cytoplasmic spots’ folder in step 34a if only the cytoplasmic mRNA output is required.
  d. Repeat steps a to c for the ‘scFv spots co-located’ and ‘scFv spots co-located inside Surfaces’ data.
34. Run the data through the spotME_EmbryoMid.py script (https://github.com/TMinchington/sass) (Figure 3, Step 6). In this script, the total mRNAs and translated mRNAs are assigned to the nearest nuclei, to reveal the numbers in each of these virtual cell territories across the expression domain.
  a. Create a folder containing the ‘SunTag mRNA spots’ data (or ‘Suntag mRNA cytoplasmic spots’ if step 33 is used) and ‘nuclei’ data.
  b. In Anaconda Prompt, type: python. Drag and drop the spotME_EmbryoMid.py script and then drag and drop the folder created in step a.
  c. The output required is the ‘all_data_with_distance.txt’ in the ‘time_data’ folder. This is the data output for all mRNAs.
  d. Repeat steps a to c with the ‘scFv spots co-located’ data instead of ‘SunTag mRNA spots’ data. This will give the data output for translated mRNAs only.
  e. The ‘all_data_with_distance.txt’ file gives the number of mRNA or translation site spots assigned to each nearest nucleus, the x, y, and z position of each spot and nucleus, and the Imaris intensity data for each spot in each channel. In addition, the script calculates the anterior-posterior axis of embryos positioned as described in Step 23. The midline.png file shows the axis position (Figure 6B). The ‘all_data_with_distance.txt’ file also shows the position of each nucleus along the anterior posterior axis, and the perpendicular distance from the axis. If the embryo axis has instead been lined up along the x or y axis, the x or y positions can be used as a measurement along the axis.

***Alternatives:*** Other free to access programs are available to recognize foci in fluorescence images, including Airlocalize (Trcek et al., 2017) and FISH-quant (Mueller et al., 2013). However, we find these are less able to cope with large datasets than Imaris.

#### Analysis of ribosome number

**Timing: ∼30 min per image**

In this step, the number of ribosomes present in the translation sites are calculated, using the high magnification images collected. This quantification requires analysis of the sum fluorescence intensities of both single cytoplasmic proteins and active translation sites. Here, we use the freely available analysis software Airlocalize that uses a spot-detection algorithm (Lionnet et al., 2011; Trcek et al., 2017) (Figure 3B). This algorithm subtracts the background fluorescence that surrounds individual mRNA spots and therefore provides robust measurements even in images containing high background.

35. Prepare the high magnification images for analysis.
  a. Open the saved Leica image file in Fiji.
  b. Save each channel collected separately as a .tif image.
  c. Name the Suntag smFISH channel as suntag.tif, and the mNeonGreen channel as ng.tif, for compatibility with later analysis scripts.

***Alternatives:*** To reduce the computational time in Airlocalize, a subset of the image can be used.

36. Identify and quantify the fluorescence of translation sites using Airlocalize.
  a. Open the Airlocalize software. When prompted select the type of data to be analyzed, here a 3D single image.
  b. Navigate to and select the image that will be analyzed.
    i. Select the mNeonGreen image channel only, named ng.tif.
  c. Determine the general analysis parameters in the next window, ‘Quantification Parameters’.
    i. Use the default settings in the ‘PSF Width (pix)’ section. This will enable the user to manually select fluorescent spots that will be used to calculate the point spread function (PSF) (see Step d).
    ii. Select an output folder.
    iii. Enable the option ‘output image of spots’. This option will output a tif image file with all the spots that were recorded during the analysis. To verify the analysis accuracy this spot file can be overlaid with the microscopy image.
  d. Determine the PSF. Airlocalize visualizes the fluorescent profile along the x/y/z axis of a selected fluorescent spot and a Gaussian fit is applied to it (Figure 3B, Step 1).
    i. Select a translation site by hovering the cursor over the center of the fluorescent signal and left click verify selection. The x/y/z profiles of this spot will be displayed. Select ‘local gaussian fit’ to display the fit over the fluorescent profile. A good fit resembles a Gaussian distribution curve. Click ‘record fit result’ to provide training data for the algorithm. Repeat with ∼ 10 additional translation sites.
    ii. Advance to the next window by selecting ‘done’.
  e. Identify the translation sites using a detection threshold. Modify the threshold until all translation sites are recognized by a red signal overlay on the microscopy image but none of the single proteins are. Once the desired threshold is set, select ‘done’ (Figure 3B, Step 2).
  f. Multiple files will be generated in the output folder.
    i. The spots.tif file contains all spots that were identified.
    ii. The .loc3 file contains the x/y/z position, fluorescence intensity and frame number (here 0) for each translation site.
    iii. The .par3 file contains all analysis parameters (including PSF in xy and z).
    iv. The .det file contains the results and parameters.
  g. Move the analysis files into a subfolder called ‘translation_sites’.
37. Identify and quantify the fluorescence of single proteins using Airlocalize (Figure 3B, Step 3).
  a. To identify single proteins, repeat steps 36a and b.
  b. To identify single proteins there are two options:
    i. Either follow steps 36c and d as outlined above
    ii. Or disable the option ‘set manually’ in the ‘PSF Width (pix)’ window. Instead modify the PSF values to the previously determined values in the translation site analysis. The PSF values are included in the .par3 text file. This option will skip step 36d and advance directly to step 36e.
  c. Follow steps 36e and f. Make sure that the threshold is set so that single proteins are recognized.
  d. Move analysis files into a subfolder called ‘single_proteins’.

***Note:*** By identifying single proteins we also by default record the translation sites again as their fluorescent intensity is greater than single proteins. However, the translation sites will later be removed from this dataset in step 39b.

38. Identify and quantify the fluorescence of mRNA molecules for colocalization analysis using Airlocalize (Figure 3B, Step 4).
  a. Follow steps 36a-f as outlined above using the SunTag mRNA microscopy channel. The input file should be called suntag.tif.
  b. Move the output files into a subfolder called ‘mRNA’.
39. Perform colocalization analysis and determine the number of ribosomes present in the translation sites.
  a. Open Anaconda prompt. Type: python. Drag and drop the folder containing the ribosome_number.py script (https://github.com/DaisyVinter/StarProtocols_Vinter_2021) and then drag and drop the folder containing the subfolders with the analysis (Figure 3B, Step 5).
  b. The script will identify translation sites by colocalization with mRNA spots, discarding any ‘translation sites’ that do not colocalize. The script will then take the single proteins and discard any included translation sites. Following this, the median fluorescence of the remaining single proteins is calculated, and the fluorescence of translation sites is divided by this number.
  c. The output will be a file called num_ribo.csv containing the number of ribosomes per translation site.
  d. Apply a correction factor to account for ribosomes that have only translated part of the Suntag mRNA sequence and so do not have the full binding of fluorophores.
    i. The correction factor is calculated by (length_SunTagCDS_ × 0.5 + length_hbCDS_)/length_total_. This assumes that ribosomes are uniformly distributed, so ribosomes that have only translated part of the Suntag sequence will on average have translated half of the sequence (Pichon et al., 2016).
    ii. Divide the number of ribosomes given in the output file in c by the correction factor calculated (Figure 3B, Step 6).

***Note:*** The Airlocalize algorithm is MATLAB based but does not require a full MATLAB license and can be run solely with the MCRInstaller.

### Section 2: Live imaging of translation sites

Here we describe how to image live embryos to visualize translation sites across the expression domain and gain spatiotemporal information about their distribution. Separate steps are also included in the protocol describing how to monitor the translation of single mRNAs in live embryos, in order to capture their translation dynamics (Figure 2).

#### Prepare cover slips for live imaging

**Timing: 2 days**

In this step, cover slips, coated thinly with heptane glue, are prepared for their use in live imaging experiments.

***Note:*** Prepare cover slips the day before or on the day of the live imaging experiment.

40. Make a ball of unrolled sellotape and place it in a 50 mL bottle. Fill the bottle half full of heptane and soak the sellotape for at least 12 h so the glue will dissolve.

***Note:*** We use yellow double-sided plastic tape, 25 mm x 20 m (RS Components, Cat# 555-033) or packaging tape (Tesa, Cat# 4124). Other brands of sticky tape will need to be tested to ensure they are not toxic to *Drosophila* embryos.

**Pause point:** Heptane glue can be kept for many months at 20^o^C.

41. Take Nr. 1, 18 x 18 mm coverslips and pipette approximately 60 μL of heptane glue onto the coverslip. Leave the heptane glue to dry as a thin coating on the slide.

#### Set up the embryo collection cage

**Timing: 1–3 days**

**Figure 4:**
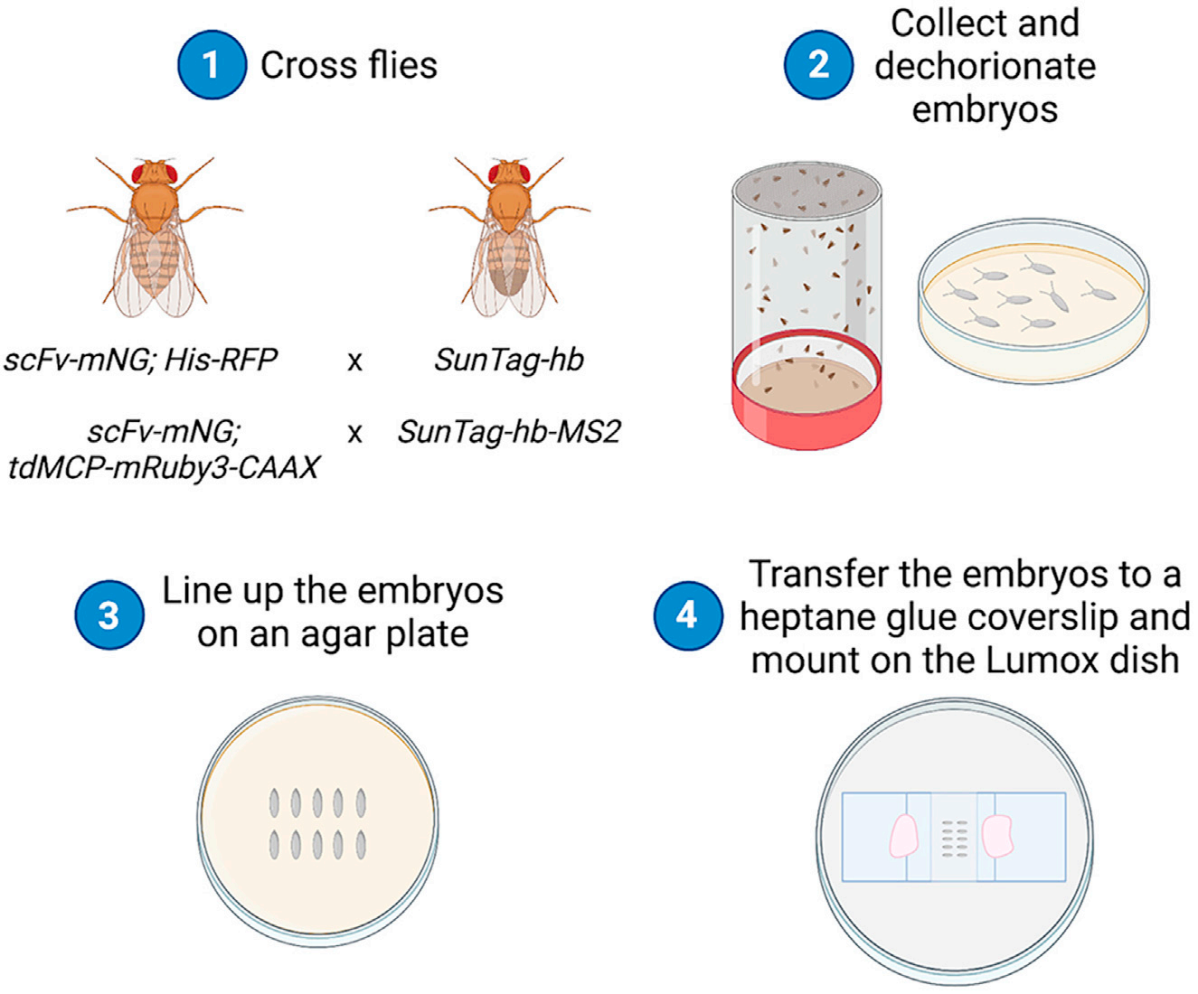
Overview of embryo mounting for live imaging of translation sites The key steps required for the collection and mounting of embryos are shown.

In this step, a collection cage is set up to produce embryos for live imaging (Figure 4, Step 1).

42. Collect flies.
  a. *For whole expression domain live imaging (Step 50):* Collect virgin females heterozygous for the *scFv-mNG* and *His-RFP* transgenes and males homozygous for the *SunTag-hb* transgene.
  b. *For single translation site live imaging (Step 51):* Collect virgin females heterozygous for the *scFv-mNG* and *tdMCP-2xmRuby3-CAAX* transgenes and males homozygous for the *SunTag-hb-128xMS2* transgene.

***Note:*** This cross will produce embryos with one copy, zygotically expressed, of the tagged gene of interest.

***Note:****nos-MCP-EGFP* (Garcia et al., 2013) and *nos-tdMCP-RFP* (Halstead et al., 2015) stocks are also available for detection of the mRNAs carrying MS2 sequences.

43. Transfer at least 50 females and 25 males to a collection cage with an apple juice agar plate and yeast.

***Note:*** Including more flies in the collection cage will give more embryos for live imaging.

#### Collect and mount embryos

**Timing: Embryo collection, ∼60 min; mounting, ∼10 min**

In these steps, embryos are mounted for live imaging (Figure 4).

44. Change the collection cage plate to a new apple juice agar plate with yeast. Let the flies lay for the desired amount of time and remove the plate for embryo collection (Figure 4, Step 2).

***Note:*** For imaging *SunTag-hb* mRNAs, which are translated from nc11 – 14, we let flies lay for 1 h so that the embryos are sufficiently young to image the whole translation time.

45. Dechorionate the embryos by applying 50% domestic bleach to the plate and incubating for 2 min.
46. Transfer the embryos to a sieve by washing them off the plate with water or embryo washing solution. When in the sieve, wash thoroughly with water or embryo washing solution until no bleach remains.
47. Brush the embryos out of the sieve onto a fresh apple juice agar plate under a dissection microscope.
48. Arrange the embryos in one or more lines on the apple juice agar plate using a brush or tweezers (Figure 4, Step 3).

***Note:*** Arrange the embryos so that the embryo area to be imaged is facing upwards. For example, the *scFv-mNG*; *His-RFP*/*SunTag-hb* embryos were arranged with the lateral side facing upwards.

49. Mount the embryos on a Lumox dish (Figure 4, Step 4).
  a. Place two Nr. 0, 18 **×** 18 mm cover slips on the Lumox imaging dish, leaving a small gap of approximately 12 mm in between.
  b. Fill the gap with 70 μL of halocarbon oil (7:1, halocarbon oil 700:halocarbon oil 27).
  c. Use tweezers to pick up a heptane glue cover slip and carefully lower the glue side onto the embryos.
  d. Transfer the coverslip with embryos onto a Lumox imaging dish, with embryos positioned in the imaging oil and the coverslip overlapping with the two coverslips placed on the imaging dish in step a.
  e. Use nail polish to secure the embryo coverslip to the other coverslips.

***Note:*** A movie of this mounting technique can be found in (Hoppe and Ashe, 2021b).

#### Live imaging of translation sites across the expression domain

**Timing: ∼ 2.5 h per video**

In this step, *SunTag-hb* mRNA translation sites are imaged across the width of the expression domain in living embryos. For precise aging of embryos, the embryos are maternally expressing His-RFP, therefore we are visualizing translation sites without detecting the mRNAs (Figure 1A). However, this has the advantage that the mRNA is only modified by the addition of SunTag sequences and does not carry any MS2 loops, meaning there is less potential for endogenous regulation to be disrupted.

50. Collect a time-lapse movie of a developing embryo for ∼2 h. We use a Zeiss LSM 880 upright microscope with Fast Airyscan for imaging (Troubleshooting 1 and 3).
  a. Set up the microscope with two channels for scFv-mNG and His-RFP, both collecting with the Fast Airyscan.
    i. For the example dataset, we used a 40**×**/1.30 EC Plan-Neofluar DIC M27 oil objective and 1.1**×** optical zoom, with a bit depth of 16. The image was acquired at 2996 x 788 pixels, resulting in a pixel size of 0.06 μm, and 25 slices with a z-step size of 850 nm.
    ii. The His-RFP channel was excited with the 561 nm laser (1.5%) and the scFv-mNG channel with the 488 nm laser (5%).
  b. Locate the embryos using brightfield illumination.
  c. Using the His-RFP channel, find an embryo of ∼15 min prior to the desired age of imaging. For imaging translation beginning at nc11, as for the *SunTag-hb* embryos, find an embryo where nuclei are just beginning to migrate to the surface.
  d. Set up a z-stack including the whole nuclear depth and cytoplasm on either side of the nucleus.
  e. Take a time series with the minimum possible gap between z-stacks until translation has ceased. An example of a *SunTag-hb* time-lapse experiment is shown in Methods video S1 (previously described in Vinter et al. 2021).

***Note:*** Speed, resolution, and z-depth of imaging must be balanced to achieve the optimal settings for the experiment. A high frame rate is important to capture the dynamics of translation, but may impact on the image quality. If raw image quality is low, image analysis and identification of translation sites above background may be more difficult. Time resolution can be improved by imaging a smaller region of the embryo, or by reducing the z-stack. The settings used allow us to see a narrow region of the embryo (∼9 nuclear widths at nuclear cycle 14) extending along ∼30% of the anterior posterior axis, with a time resolution of ∼40s and imaging depth of 20.4 μm. The resolution allows for the recognition of individual translation sites in Imaris. If using a different microscope or imaging a different sized section of the embryo, these settings will need to be optimized.

Methods video S1. Maximum intensity projection of an embryo showing *SunTag-hb* translation sites (gray) and Histone-RFP (magenta) through nc11-14, related to step 50 Embryos zygotically expressing *SunTag-hb* are from females carrying single copies of the*scFv-mNG*and*His-RFP*transgenes. Imaged with 46 sec time resolution between frames, time stamp is in min:sec.

#### Live imaging of mRNAs and single translation sites

**Timing: ∼30 min per video**

In this step, *SunTag-hb* mRNAs and translation sites are imaged in a small region of the expression domain in live embryos. As mRNAs move very rapidly, we are using tdMCP-2xmRuby3-CAAX to tether them to the membrane, thereby facilitating their tracking and analysis over time (Figure 1C).

51. Collect a high magnification time-lapse dataset of individual translation sites. We use a Zeiss LSM 880 upright microscope with Fast Airyscan for imaging (Troubleshooting 3).
  a. Set up the microscope with two channels for scFv-mNG and tdMCP-2xmRuby3-CAAX, both collecting with the Fast Airyscan.
    i. For the example dataset, we used a 63**×**/1.40 PL Apo oil objective and 8**×** optical zoom, with a bit depth of 16. The image was acquired at 184 **×** 184 pixels, resulting in a pixel size of 0.09 **×** 0.09 μm.
    ii. The MCP-mRuby3 channel was excited with the 561 nm laser (15%) and the scFv-mNG channel with the 488 nm laser (15%).
  b. Locate the embryos using brightfield illumination.
  c. Locate the area where translation is occurring or expected to occur using the objective without zoom (Troubleshooting 4).
  d. Zoom into the area so that only a few cells are visible.
  e. Adjust the focus so that the membrane (labeled with tdMCP-2xmRuby3-CAAX) is in focus, with individual translation sites in mNG if the mRNAs are already being translated (Troubleshooting 5).
  f. To achieve the best possible time resolution, a time lapse dataset was acquired with a single z plane. An example time-lapse experiment imaging individual translation sites is shown in Methods video S2.

***Note:*** Prior to nc14, membranes are only present apically in the embryo, then membrane invagination starts in nc14. Therefore, when imaging prior to nc14, it is necessary to focus on the top of the cells where the mRNAs and unbound tdMCP-mRuby3-CAAX proteins show the cell outline (Figure 7C). Once the membranes are located zoom in to image the translation sites.

***Note:*** To track single translation sites, speed, resolution, and z depth must be balanced. In the example data set, a single z plane and small region was used so that the image resolution was of the quality necessary to identify translation sites (pixel size 0.09 **×** 0.09μm), and the time resolution was ∼2s, which is fast enough to capture changes associated with translation initiation and elongation (Morisaki and Stasevich, 2018). When using a different microscope, settings will have to be adjusted to optimize for each individual experiment.

Methods video S2. Single plane time-lapse experiment visualizing membrane tethered tdMCP-mRuby3-CAAX and bound *SunTag-hb-MS2* mRNAs (magenta) and translation sites bound by scFv-mNG (green), related to step 51 Embryos are from females carrying single copies of the*scFv-mNG*and*tdMCP-Ruby3-CAAX*transgenes crossed to*SunTag-hb-MS2*transgenic males. Merged and single channel views are shown. Time resolution is 1.98 sec per frame, the scale bar is 5 μm.

#### Analysis of expression domain live imaging

**Timing: 1 h**

**Figure 5:**
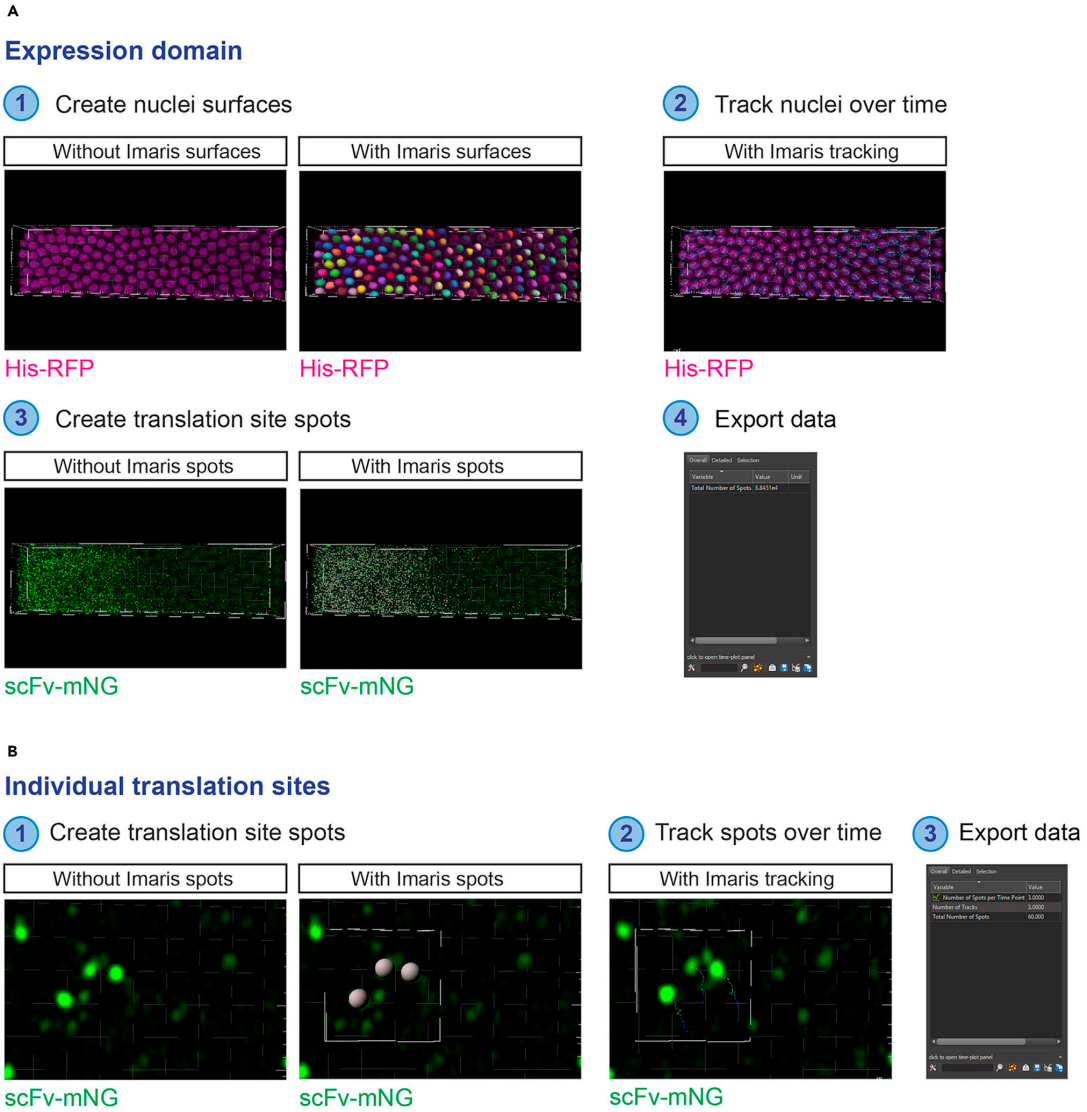
Overview of the analysis of translation sites from the live imaging data The analysis steps are shown for live imaging of (A) the expression domain and (B) anchored individual translation sites.

These steps describe how to analyze the expression domain level live imaging data to reveal the number, intensity, and distribution of translation sites over time (Figure 5A).

52. Using the Imaris ‘surface’ function, create surfaces to identify nuclei (Figure 5A, Step 1).
  a. In the first screen, enable ‘Track Surfaces (over Time)’.
  b. Select the Channel used for His-RFP.
  c. Select ‘Enable Smooth’ and set ‘Surfaces Detail’ to 1 μm, which will smooth the nuclear surface and aids object identification.
  d. In the ‘Slice’ tab, measure the diameter of nuclei.
  e. Select ‘Background Subtraction’ under Thresholding. Set ‘Diameter of largest Sphere which fits into the Object’ to the value measured in step d.
  f. Adjust the threshold so that the nuclei are selected. Move between frames to check that nuclei are selected in all frames.
  g. Select ‘Enable’ under ‘Split touching Objects (Region Growing)’. Set ‘Seed Points Diameter’ to the value measured in step d.
  h. Filter the seed points by ‘Quality’ so that only nuclei are selected.
  i. Use the ‘Number of Voxels Img=1’ filter to eliminate any small non-nuclear objects.
  j. In the ‘Tracking’ screen, select the Algorithm as ‘Autoregressive Motion’, Max Distance as 5 μm, and Max Gap Size as 3. These settings work well to track nuclei (Figure 5A, Step 2) from nc11 to the onset of gastrulation in nc14, where nuclei do not move more than 5 μm between consecutive frames. If analyzing datasets with longer time resolution, determine the maximum nuclear movement between two consecutive frames using the ‘Slice’ tab.
  k. Complete the surfaces wizard.
53. Using the Imaris ‘spots’ function, create spots to identify the translation sites (Figure 5A, Step 3) (Troubleshooting 2).
  a. Deselect ‘Track Spots (over Time)’ in the first screen.
  b. Select the Channel used for ‘scFv-mNG’.
  c. In the ‘Slice’ tab, measure the size of the translation sites.
  d. Enter the value into ‘Estimated XY diameter’ and select ‘Model PSF-elongation along Z-axis’ and ‘Background Subtraction’.
  e. Use filters to select the scFv-mNG foci (translation sites). Useful filters include ‘Quality’, ‘Intensity Max’ and ‘Intensity Sum’. Move between frames to check foci are selected in all frames.
  f. Complete the spots wizard.
54. Export the Imaris data (Figure 5A, Step 4).
  a. Export the statistics in the ‘Statistics’ tab. Select the desired statistics in ‘Preferences’, which can include the scFv-mNG intensity of each spot and position of the spots in each time point.

#### Analysis of single translation site live imaging

**Timing: 30 min**

In this step, individual translation sites are tracked, and their fluorescence intensities quantified over time.

55. Use the Imaris ‘Spots’ function for tracking of translation sites over time (Figure 5B) (Troubleshooting 6).
  a. In the creation wizard select ‘Track spots over time’.
  b. In the next window, select the scFv-mNG channel for analysis and estimate the xy diameter of the translation site. The full fluorescence of the translation site should be included within the spot object (Figure 5B, Step 1).
  c. Using filters, make sure that the translation sites are selected through time. The most useful filters here are ‘Quality’, ‘Intensity Sum’ and ‘Intensity Max’.
  d. For the tracking parameters select ‘Autoregressive Motion’, a ‘Maximum Distance’ of 5 μm and a ‘Max Gap Size’ of 2 (Figure 5B, Step 2).
56. After successful tracking, export the analysis files.
  a. In the ‘Statistics’ tab, select ‘Export all Statistics to file’. The statistics that are exported can be defined by the user under settings and are dependent on the type of analysis required. These can include (but are not limited to) fluorescence intensity quantification, distance and speed of movement (Figure 5B, Step 3).

***Note:*** For fluorescence intensity quantitation it is important to perform a background correction. This can be achieved by manually placing spot objects (of identical volume to the analysis spots) into different background regions and measuring the fluorescence value. An average background value can then be subtracted from the translation site fluorescence. Depending on the movie length this can be time consuming. For a semi-automated way to remove background fluorescence see (Hoppe and Ashe, 2021b).

## Expected outcomes

**Figure 6:**
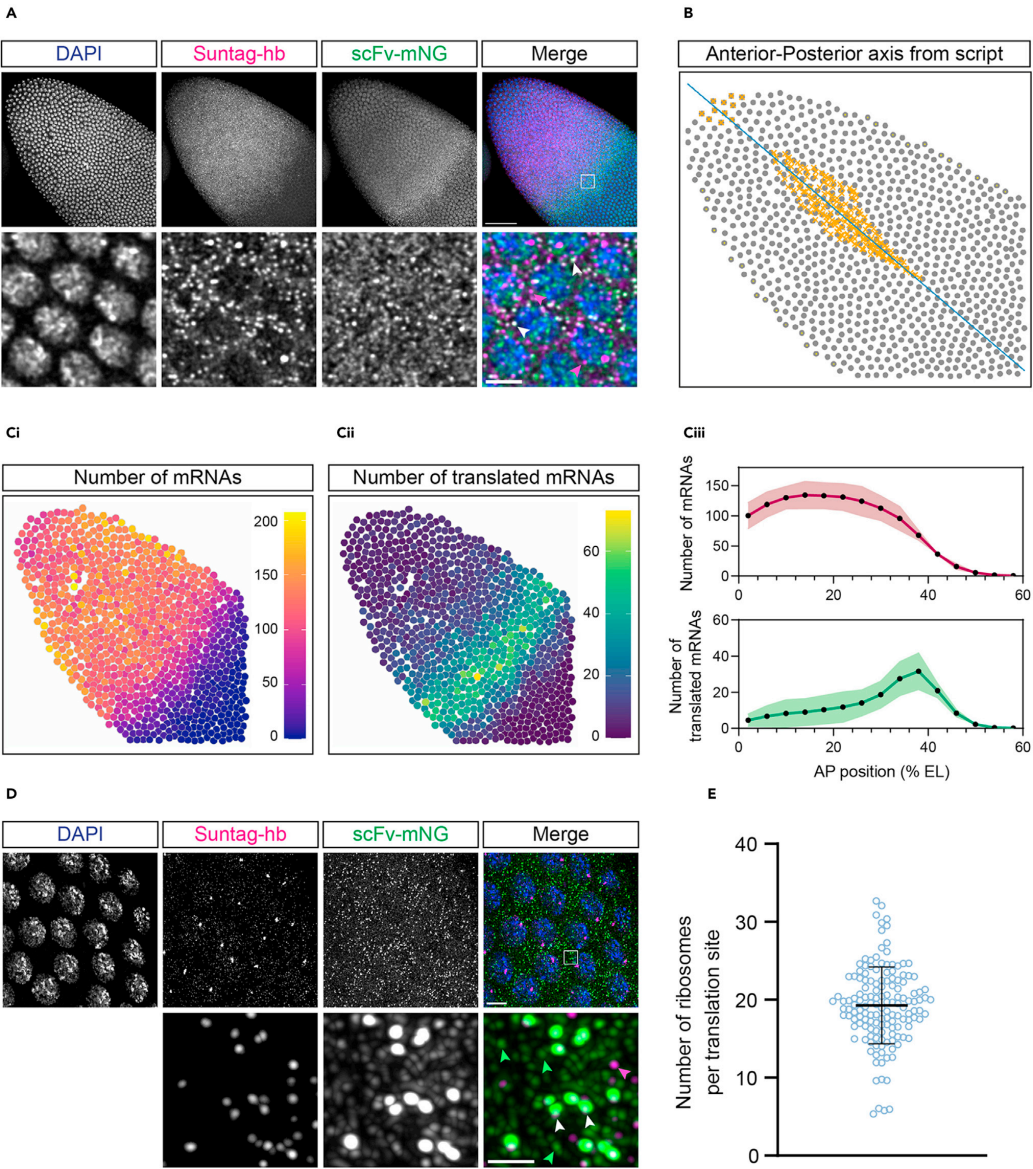
Expected outcomes from the fixed imaging of translation sites (A) Image of the whole expression domain of a nuclear cycle 14 embryo expressing *scFv-mNG* and *SunTag-hb*, stained with smFISH probes for SunTag (magenta), anti-mNG antibody (green) and DAPI (blue). The lower panel shows a zoomed inset of the image, the position is shown by a box. The bright signals in the scFv-mNG channel, colocalizing with mRNAs, are translation sites (white arrowheads), the dimmer signals are single proteins and background signals. The weaker magenta signals (pink arrowheads) in the merged image represent untranslated mRNAs, the bright magenta signals are transcription sites. Upper panel, scale bar = 50 μm. Lower panel, scale bar = 5 μm. (B) Output of the spots assignment script showing the calling of the AP axis (blue line). The nuclei highlighted at the anterior are used to find the axis. (C) (i) The embryo is false colored by the number of mRNAs per nuclear territory as outputted by the spot assignment script. (ii) As in (i), but for the number of translated mRNAs. (iii) Graphs show the number of mRNAs or translated mRNAs per nuclear territory along the AP axis. Nuclear territories are binned along the AP axis (bin size = 20 μm) and the mean value per bin is determined. n = 3 embryos, mean ± SD is shown. (D) Image of a nc13 embryo, expressing *scFv-mNG* and *SunTag-hb*, taken for quantification of the number of ribosomes in translation sites. The lower panel is a zoomed inset of the image, marked by a white box, showing bright translation sites (green) colocalized with mRNAs (magenta) and dimmer single proteins (green). White, green and pink arrowheads mark translation sites, single proteins and untranslated mRNAs, respectively. The bright signals in the *SunTag-hb* channel in the top image are transcription sites. Upper panel, scale bar = 5 μm. Lower panel, scale bar = 1 μm. (E) Ribosome number quantification for the region of interest from (D). n = 146 translation sites from 1 nc13 embryo, mean ± SD shown.

The smFISH/immunofluorescence of fixed embryos will allow the total SunTag-POI mRNAs to be visualized; colocalization of the scFv-mNG signal will indicate that they are being translated (Figure 6A shows the data for *SunTag-hb* mRNAs). For each embryo, the total number of mRNAs per nuclear territory, the number of translated mRNAs per nuclear territory, and data relating this to position along the anterior-posterior axis can be outputted (Figure 6C). As part of the analysis, the number of total and translated mRNAs can also be visualized spatially for each cell in the expression domain using a heatmap color scale (Figure 6C). At nc13, the total number of *SunTag-hb* mRNAs declines towards the center of the embryo, but the proportion translated is constant (Vinter et al., 2021). However, we found altered translation dynamics at nc14, as translation of *SunTag-hb* mRNAs is restricted to only a stripe of cells at the posterior edge of the expression domain (Figures 6A and 6C) (Vinter et al., 2021). The data can also be analyzed to determine the ribosome number in each translation site (Figures 6D and 6E). Ribosome number can also be determined from live imaging data based on the relative fluorescent signals of translation sites versus single proteins (Pichon et al., 2016; Yan et al., 2016), if the latter can be reliably detected.

**Figure 7:**
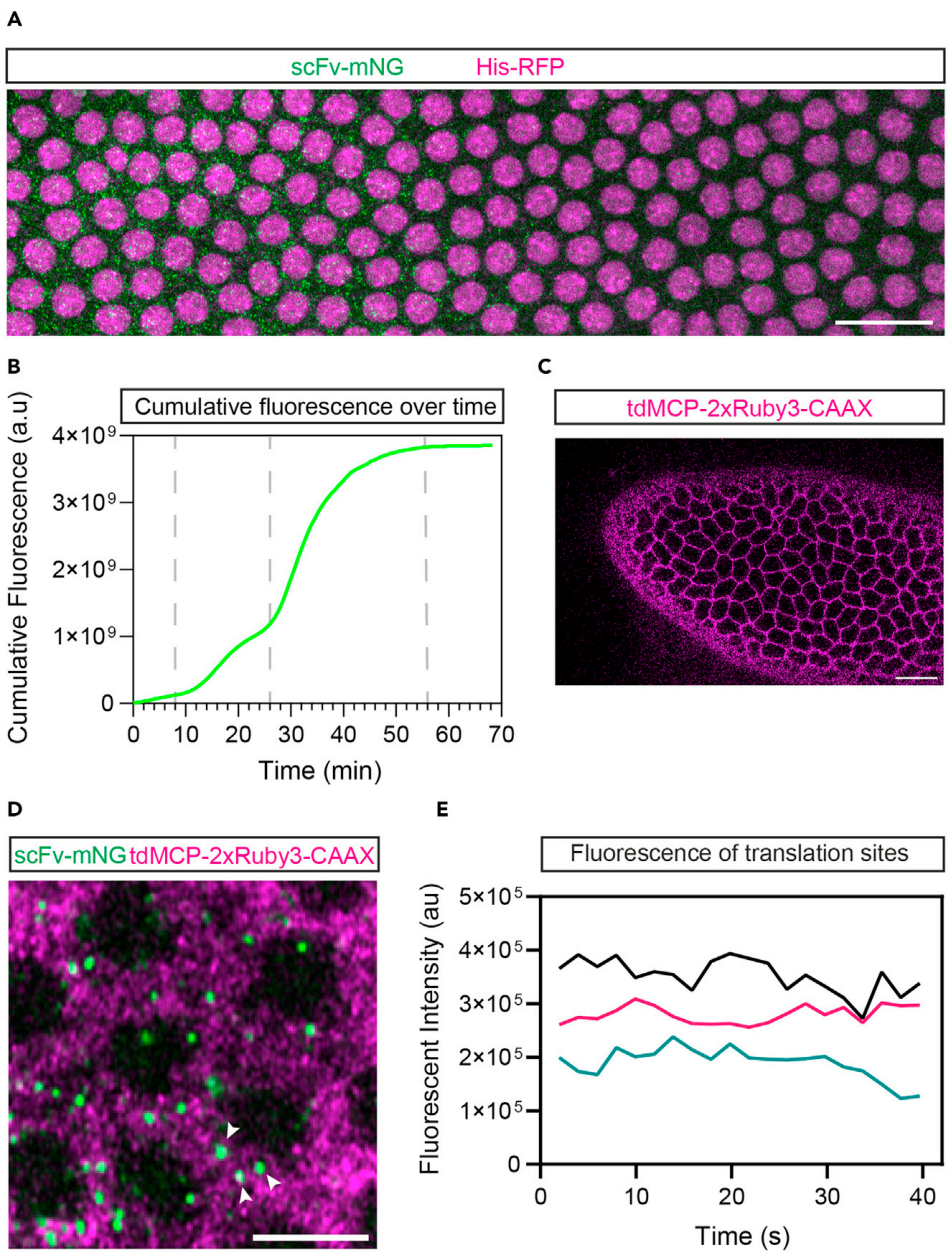
Expected outcomes from the live imaging of translation sites (A) Still from Movie 1 of the expression domain at mid nc13 in an embryo expressing *Histone-RFP*, *scFv-mNG* and *SunTag-hb*, showing nuclei (magenta) and translation sites (green). The embryo is oriented with anterior to the left. Scale bar = 20 μm. (B) Example graph of cumulative translation over time (in fluorescence arbitrary units) in the embryo shown in Movie 1. The vertical bars mark the start of nc12, 13 and 14. (C) Still from a movie showing that the tethered tdMCP-2xRuby3-CAAX proteins label membranes. Scale bar = 20 μm. (D) Still from Movie 2 of individual translation sites in an embryo expressing *scFv-mNG*, *tdMCP-mRuby3-CAAX* and *SunTag-hb-MS2*, showing mRNAs and membranes (magenta) and translation sites (green). The bright magenta foci are likely individual mRNAs. Arrowheads point to the translation sites used for fluorescence quantification in (E). Scale bar = 5 μm. (E) An example graph of fluorescent traces of individual translation sites from the movie in (D).

The data on the single cell efficiency of translation determined by the fixed embryo analysis is complemented by the live imaging data that provide temporal information about translation (Figures 7A and 7B, Methods video S1). For example, live imaging of the *SunTag-hb* translation sites across the expression domain reveals that the domain of translated *SunTag-hb* mRNAs gradually contracts from the anterior of the embryo starting at late nc13, so that only a stripe of translation remains in early nc14 embryos (Vinter et al., 2021).

Live imaging of single translation sites allows the translation of individual mRNAs to be followed over time (Figures 7D and 7E, Methods video S2). Imaging at this resolution allowed us to capture different events in the translation cycle, including release of a newly synthesized protein and translation termination (Vinter et al., 2021).

## Limitations

When live imaging the whole expression domain using the method described in this protocol, it is not possible to resolve single mRNAs as well as translation sites, as MS2 loops are not included. Use of MS2 loops, an MCP-FP and His-FP in a third channel (for example, His2Av-eBFP2 (Fukaya et al., 2017)) may allow this, but we have not tested this. In addition, weaker translation sites with less ribosomes and therefore less fluorescence may be missed. While live imaging can give temporal information about translation, it is unable to show the proportion of mRNAs translating, as untranslated mRNAs cannot be visualized on a large scale. Fixed imaging with smFISH to visualize single mRNAs gives information about the number of translated and untranslated mRNAs, however it cannot give information about changes over time. Therefore, the fixed and live approaches are complementary and allow different information about translation to be collected.

Live imaging and tracking of single translation sites are limited by the time resolution that can be achieved by the microscope used, and the length of time single translation sites can be followed due to movement out of the frame of imaging or bleaching. These limitations will be highly dependent on the microscope system and settings used. In addition, we use a CAAX tag in the MCP protein to anchor translating mRNAs to the plasma membrane. Although this method allows for longer tracking times, it is possible that mislocalizing the translating mRNA in this way may affect the regulation of translation. Many mRNAs in *Drosophila* embryos are subcellularly localized (Lecuyer et al., 2007), and recent data has shown that translation efficiency of some mRNAs is dependent on their localization (Dufourt et al., 2021). We recommend that fixed imaging without the MCP protein is always carried out to check that translation is not dependent on the subcellular localization of the specific mRNA that is being investigated. Finally, insertion of the MS2 sequences into the 3′UTR may disrupt translational regulation. However, this can be assessed by comparing the translation efficiency of the test mRNAs with and without any MS2 loops in fixed embryos.

## Troubleshooting

### Problem 1

Translation sites cannot be visualized when imaging fixed (Step 23) or live (Step 50) embryos.

### Potential solution

We find that the microscope settings described work robustly for the *SunTag-hb* and *scFv-mNG* fly lines. If a different tagged mRNA of interest is investigated, it may be weakly translated, which would decrease the brightness of the translation site fluorescent signal. Try increasing the laser power used for the scFv channel.

### Problem 2

Non-translation sites are detected as spots in the Imaris analysis of the fixed (Step 28) and live (Step 53) embryo data.

### Potential solution

Imaris may incorrectly identify background scFv signal (either free scFv-FP or SunTag proteins that have been bound by scFv-FP and then diffused away) as spots, in both fixed and live imaging. In fixed imaging, these non-translation site spots will be removed by the process of colocalizing scFv spots with SunTag mRNAs, as only translation sites will be colocalized with mRNAs. In this case, use the Imaris thresholds that capture all visible translation sites, ignoring any non-translation site spots that are identified. It is possible to check that known non-translation site spots are removed by comparing the original scFv spots mask with the scFv spots co-located mask.

If Imaris recognizes background noise rather than bright fluorescent foci in live imaging, adjust the ‘Quality’ and ‘Intensity Sum’ filter thresholds so that these spots are eliminated. It may be that, for different time points in the movie, different thresholds are necessary to recognize all translation sites and eliminate background noise spots. In this case, divide the time series and set the thresholds separately, then combine using the Imaris ‘Merge’ function. If single translation sites are tracked over time, it is possible to perform the spot identification using different thresholds and merge them into one spot object file prior to tracking.

A smoothing wavelet filter Imaris X-tension (available at https://github.com/zindy/Imaris) can also be used on the scFv-mNG channel to remove background noise. Spots can then be detected using this channel, and statistics exported for the fluorescence of the original scFv-mNG channel so the original fluorescence is reported.

### Problem 3

Developmental defects occur when live imaging (Steps 50 and 51).

### Potential solution

Developmental defects may occur due to stress on the embryo. Check that the temperature in the room is not above 27°C, and do not attach the coverslip with too much pressure. In addition, developmental defects occur at a higher frequency if the flies used for embryo collection are older. If the cage used is over 10 days old, collect fresh virgin females and males and set up a new collection cage.

### Problem 4

The developmental stage or position in the embryo cannot be identified when imaging single translation sites (Step 51c).

### Potential solution

As His-RFP is not used for crosses where the aim is to image single translation sites, it can be difficult to identify the position in the embryo or embryonic stage, especially at higher zoom. Try viewing the embryo in brightfield illumination, which will show if embryos have cellularized or gastrulated. Membranes should be visible in the tdMCP-2xmRuby3-CAAX channel at the top of the cell, as well as some nuclear signal in the scFv-mNG channel (particularly before translation has begun), as unbound scFv-mNG is localized to the nucleus. Use these channels at low zoom to identify when the embryo has reached an appropriate age and zoom in to the region of interest of the expression domain. In addition, His2Av-eBFP2 fly lines are available (Fukaya et al., 2017), which could be introduced to allow nuclei to be imaged.

### Problem 5

Cannot find mRNAs or translation sites when live imaging a high magnification region of the embryo (Step 51e).

### Potential solution

Ensure the experiment is set up so that the membrane is in focus, as this is where translating mRNAs will be tethered. We find it easier to focus on tethered mRNAs to set up the imaging experiment. In our experience it is harder to keep single mRNAs in focus than their bulkier translation sites. In addition, the cell membranes become very thin shortly before and during mitosis, making it easier to follow individual mRNAs. Note that there is high tdMCP-mRuby3-CAAX signal at the membrane based on concentration of the tdMCP-mRuby3-CAAX proteins, even in the absence of bound mRNAs.

### Problem 6

Tracking is unsuccessful for individual translation sites (Step 55).

### Potential solution

If the spots function tracking does not work well, the translation site can be tracked manually. In the first creation wizard window select ‘Skip automatic creation, edit manually’. In the ‘Edit’ tab, select ‘Auto-connect to selected Spot’ and ‘Enable delay before auto-advancing’ in the ’Manual Tracking’ section. In the first frame to be used for tracking, manually add a spot (pointer in the ‘select’ mode, press shift and left click). After placing the first spot, the software will advance automatically to the next time frame. Place the next spot until you reach the last frame that should be used for tracking.

## Resource availability

### Lead contact

Further information and requests for resources and reagents should be directed to and will be fulfilled by the lead contact, Hilary Ashe (hilary.ashe@manchester.ac.uk).

### Materials availability

The fly lines used in this protocol are available upon request from the lead contact.

## Data Availability

Example data sets have been deposited to Mendeley Data: https://doi.org/10.17632/3h73rbvbt9.1 and https://doi.org/10.17632/5vs879zvz9.1. The analysis scripts are available at GitHub: https://github.com/TMinchington/sass and https://github.com/DaisyVinter/StarProtocols_Vinter_2021. Any additional information required to reanalyze the data reported in this paper is available from the lead contact upon request.
